# Trajectory Modelling to Assess Trends in Long-Term Readmission Rate among Abdominal Aortic Aneurysm Patients

**DOI:** 10.1155/2018/4321986

**Published:** 2018-10-21

**Authors:** Ahsan Rao, Alex Bottle, Collin Bicknell, Ara Darzi, Paul Aylin

**Affiliations:** ^1^Dr Foster Unit, Department of Public Health, Imperial College London, 3 Dorset Rise, London EC4Y 8EN, UK; ^2^Department of Surgery, Imperial College London, St. Mary's Hospital, Praed Street, London W2 1NY, UK

## Abstract

**Introduction:**

The aim of the study was to use trajectory analysis to categorise high-impact users based on their long-term readmission rate and identify their predictors following AAA (abdominal aortic aneurysm) repair. Methods. In this retrospective cohort study, group-based trajectory modelling (GBTM) was performed on the patient cohort (2006-2009) identified through national administrative data from all NHS English hospitals. Proc Traj software was used in SAS program to conduct GBTM, which classified patient population into groups based on their annual readmission rates during a 5-year period following primary AAA repair. Based on the trends of readmission rates, patients were classified into low- and high-impact users. The high-impact group had a higher annual readmission rate throughout 5-year follow-up. Short-term high-impact users had initial high readmission rate followed by rapid decline, whereas chronic high-impact users continued to have high readmission rate.

**Results:**

Based on the trends in readmission rates, GBTM classified elective AAA repair (*n*=16,973) patients into 2 groups: low impact (82.0%) and high impact (18.0%). High-impact users were significantly associated with female sex (*P*=0.001) undergoing other vascular procedures (*P*=0.003), poor socioeconomic status index (*P* < 0.001), older age (*P* < 0.001), and higher comorbidity score (*P* < 0.001). The AUC for c-statistics was 0.84. Patients with ruptured AAA repair (*n*=4144) had 3 groups: low impact (82.7%), short-term high impact (7.2%), and chronic high impact (10.1%). Chronic high impact users were significantly associated with renal failure (*P* < 0.001), heart failure (*P* = 0.01), peripheral vascular disease (*P* < 0.001), female sex (*P* = 0.02), open repair (*P* < 0.001), and undergoing other related procedures (*P*=0.05). The AUC for c-statistics was 0.71.

**Conclusion:**

Patients with persistent high readmission rates exist among AAA population; however, their readmissions and mortality are not related to AAA repair. They may benefit from optimization of their medical management of comorbidities perioperatively and during their follow-up.

## 1. Introduction

The readmission rate among patients undergoing vascular procedure is high. It ranges from 8.9% to 24.4% [1, 2]. Specifically, the readmission rate after AAA (abdominal aortic aneurysm) repair varies from 12.5% to 23.2% [3, 4]. The Centre for Medicare and Medicaid Services (CMS) has reported vascular procedures to be one of the top 7 conditions that cause potentially preventable readmissions [2]. There is a possibility that, in the future hospitals, higher emergency readmission rates vascular procedures will be penalised by reduction in their reimbursement payments [2].

Limited is known about the subgroup of vascular surgery patients with higher readmission rate, also known as high-impact users [5]. They are a small proportion of the total patient population but their resource consumption is significantly high. There is not a standard and robust methodology to model and visualise changes in the frequency of long-term readmission rate in this subgroup [3, 6]. Earlier models perform poorly to predict those patients with high readmission rate and do not focus to identify predictors among high-impact users. In the previous studies, based on arbitrary classification, patients were divided into low- and high-impact groups [7]. Recent evidence suggests that subgroups other than low- and high-impact exist when the patient population is followed up for a longer period of time [8, 9]. In a current study, 5 subgroups of patients were identified based on the pattern of recovery following stroke, each with distinct prognosis and risk of mortality [10].

Group-based trajectory modelling (GBTM) has advantages over other models used to study longitudinal data [11]. The expected trajectory of each subgroup is based on repeated observations over time. It assumes that the subgroups are part of the same population but each follow different developmental pathways. GBTM can be used to study both continuous and binary outcomes by incorporation of various statistical methods [11]. It does not pre-empt the number of groups but uses the statistical device for approximating the unknown distribution of trajectories across the population [11]. Although GBTM has only recently been used in medical research, it has been an effective tool in the analysis of longitudinal data in psychology, criminology, and social sciences over the last 25 years [6].

The aim of the study was to apply trajectory modelling on population-based data to visualise trends of readmission rate among high-impact users and other groups and to identify predictors associated with AAA surgical patients with high readmission rates.

## 2. Methods

### 2.1. Hospital Administrative Data

Hospital Episode Statistics (HES) data was used to extract information on patients diagnosed with AAA repair [12]. The data are collected quarterly by the Department of Health, Government of England, and includes information on all the inpatient hospital admissions of all the patients admitted to public hospitals in NHS (National Health Service), England [12]. All patients, including private ones, that require emergency treatment are initially admitted in these hospitals. Each hospital admission is recorded as a “spell” consisting of “episodes” which denotes the care under each consultant during the patient's stay [13]. If a hospital admission requires a transfer to another hospital before the patient is discharged, then the whole hospital stay is recorded under “superspell” [13]. For the analysis, the information on each patient's spell or superspell was retrieved. Each patient is provided a unique anonymous identifier that was used to find the patient cohort to produce longitudinal data series. The primary diagnosis of the spell is the main condition treated during the hospital stay after all investigations, diagnostic examinations, and procedures have been carried out. It accounts for the majority of the length of stay in the hospital [13]. All the conditions are coded using ICD-10 classification (International Classification of Diseases version 10). The procedures are coded using OPCS 4.7 (Office of Population Censuses and Surveys Classification of Interventions and Procedures version 4.7) [13]. The ethical approval to access the data was obtained from HSCIC (Health and Social Care Information Centre).

### 2.2. Patients with AAA Repair

All adult patients over the age of 18 who had primary AAA repair from the year 2006 to 2009 were included in the study. Patients who died during the follow-up period were included in the study as well. The statistical model used has the ability to adjust and estimate trajectory of these patients based on observation of their previous readmission rate. There were two patient cohorts: EVAR (endovascular aneurysm repair) and open repair. Initially, specific ICD-10 codes were used to identify AAA patients, as used in previous studies: elective AAA (I714, I719) and ruptured AAA (I713, I718) [14, 15]. Afterwards, the type of repair of AAA was recognised using OPCS 4 codes, as used in earlier studies, and combined with ICD-10 codes to select the patient cohort [16, 17]. The following OPCS codes were used: open repair (L18x, L19x, L20x, L21x, and L25x) and EVAR (L26x, L27x, and L28x).

For the validation of the model, the identified risk factors associated with the high-impact users were applied to a new cohort of AAA patients treated in the year 2003-2004. They were also followed up for 5 years. This was a different cohort of patients who were diagnosed with AAA a year before original cohort and followed up for similar period, providing adequate comparison data to validate the model [11].

### 2.3. Statistical Analysis

GBTM was used to categorise patients into different subgroups. “*Proc Traj*” software in SAS program was used to conduct the analysis. The outcome was the annual number of emergency readmissions for each patient for each successive year during the 5-year follow-up. In order to determine the optimum number of subgroups within a population, the choice of model was based on the following criteria: smallest value of Bayesian Information Criteria (BIC), largest value for average posterior probability for each group, odds of correct classification (OCC) > 5, and each trajectory with significant parameter estimates (*P* < 0.05). These criteria are usually chosen to test for the model with best estimate of number of groups and predictors associated with them [11, 18, 19]. BIC is based, in part, on the likelihood function to measure the efficiency of the model to predict different groups in the data. Each individual is given a probability score for one's membership in the group. For each group, the mean of the probability scores of the individuals in the group is calculated and used as an indicator for adequate internal reliability if the value is more than 0.7 [6]. Odds of correct classification measure how improved the membership probability of individuals belonging to the in-group is as compared with other groups.

The group with highest annual readmission rates was labelled as high-impact users and those with persistently lowest use of readmission rate were categorised as low-impact users. The multinomial logistic regression model was used to assess the impact of covariates on the probability of group membership while controlling for other confounding factors [11]. The group with persistently lowest use of hospital care, low-impact users, was used as a reference group. The association of each covariate was measured as the odds ratio of the impact of that covariate on the probability of membership in the specified group relative to the stable low-impact group. The covariates assessed in this study were adapted from previous clinical studies [1, 4, 20]. Transluminal angioplasty/stenting, treatment of aneurysmal segment, and bypass procedures of mesenteric, upper limb, and lower limb vessels were included in the covariate of other vascular procedures. The category of other related operations included bowel resection, revision of procedure, fasciotomy, and amputation. Charlson comorbidity score was used to assess cumulative effect of past medical problems on the patient [20]. The administrative data from the hospitals did not have information on the community and social factors, so they were not included in the analysis.

The sensitivity and specificity of the model to detect high-impact users were assessed by comparing with those patients who were actually observed to have high readmissions annually, that is, 90^th^ percentile of the number of readmissions, for 3 or more years during the follow-up period [7].

The predictability of the model was also assessed by its application to a different cohort of patients with the same condition diagnosed in the financial year before the original cohort of patients and area under curve (AUC) using c-statistics was calculated.

The methodology of the study has been reported in line with the STROCSS criteria [21].

## 3. Results

There were a total of 21,117 patients who had AAA repair. 16,973 cases were done as elective procedures, and 4144 had repair for ruptured AAA. 14,682 cases had open repair for AAA, while 6435 cases had endovascular repair.

### 3.1. Elective AAA Repair

**Figure 1:**
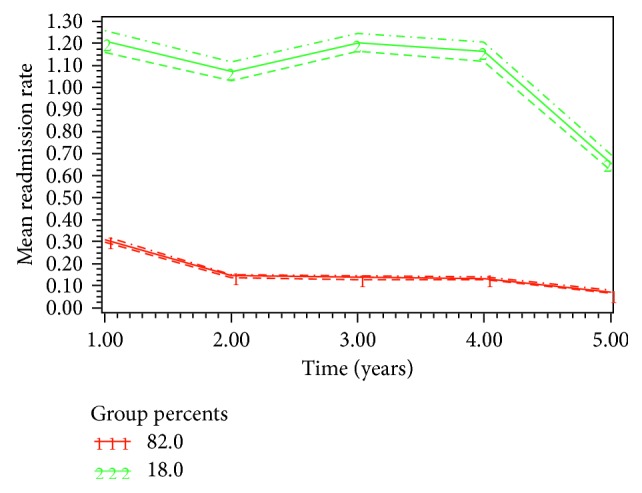
Subgroups among elective AAA repair patients and their trajectories of unplanned readmission rates (the horizontal axis starts with annual readmission rate at year one, and the dotted lines represent 95% confidence intervals for each subgroup).

The best-fit model (BIC −61509, AIC −61474) classified the patient population (*n*=16,973) into 2 groups based on their nonelective readmissions: Group 1 (82.0%) and Group 2 (18%) (Figure 1). Group 1 had persistently low rate of readmission and therefore was classified as low-impact; whereas, Group 2 had constant high rate of readmission and was labelled as high-impact. The covariates with positive and significant association with high-impact users (Group 2) were female sex, undergoing other vascular procedures, poor socioeconomic status, older age, and higher comorbidity score (Table 1). The covariates with lower odds to be related to high-impact users were chronic peripheral vascular disease, renal failure, open repair, non-Caucasian ethnicity, heart failure, and cardiac arrest. The 5-year mortality among high-impact users was 27.0% (*n*=768) vs. 23.7% (*n*=3354) for low-impact users (*P* < 0.001). The high-impact users had increased number of elective admissions during the follow-up period (mean 4.4 (SD 5.4)) compared to low-impact users (mean 2.5 (SD 3.9), *P* < 0.001). They had higher number of elective vascular procedures (mean 0.23 (SD 0.64)) compared to low-impact users (0.13 (SD 0.47), *P* < 0.001). Similarly, the high-impact group had higher number of patients undergoing revision of procedure (*n*=151, 5.3%) as compared to low-impact group (*n*=354, 2.5%, *P* < 0.001). The sensitivity and specificity of the model to detect high-impact users was 100% and 89.2%, respectively. The predictors that were significantly associated with high-impact users were applied to a different cohort of patients, validation set, who were diagnosed in the year 2003-2004 for validation of the model. The AUC for c-statistics was 0.84. In majority of the cases, the 5-year mortality was not related to AAA. The common causes of mortality were respiratory tract infection (*n*=2789, 16.4%), cancer (*n*=2512, 14.7), heart failure (*n*=1651, 9.7%), and renal failure (*n*=1201, 7.1%).

The number of patients undergoing open repair in high-impact users were higher than 57.6% of the patient population had emergency admissions (*n*=9791). Most common causes of readmissions over 5-year period were not related to AAA repair. Emergency admissions due to AAA graft complications were only 4.7% (*n*=465). AAA graft complications included leak, thrombosis, infection, and migration of the stent. Other common causes of readmissions were respiratory tract infection (*n*=748, 7.7%), chest pain (*n*=543, 5.6%), gastrointestinal haemorrhage (*n*=462, 4.7%), and external injuries (*n*=461, 4.7%).

### 3.2. Sensitivity Analysis of Elective Repair of AAA

The patient cohort of elective AAA repair was further divided into open and EVAR. GBTM was run on these 2 groups, and high-impact users among patients with open repair and EVAR were analysed.

**Figure 2:**
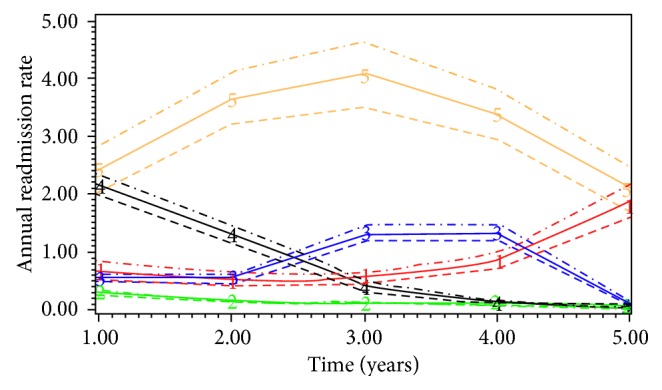
Trajectory pathways of subgroups of patients with elective EVAR repair (the horizontal axis starts with annual readmission rate after one year, and the dotted lines represent 95% confidence intervals for each subgroup).

Among elective EVAR patients (*n*=6172), the mean age of the patients was 75.1 (SD 7.1) years and mean LOS was 6.8 (SD 8.4) days. Only 0.4% (*n*=23) of the patients lived alone, and 0.3% (*n*=21) of them were discharged to nursing home. The 30-day and 5-year mortality rate was 2.3% (*n*=143) and 24.9% (*n*=1539), respectively. GBTM (BIC −24605, AIC −2453) classified the patient population into 5 subgroups based on their nonelective readmissions: low-impact (Group 2, *n*=4305, 66.0%), 2 intermediate groups (Group 3, *n*=848, 14.5%; Group 1, *n*=325, 5.9%), short-term high-impact (Group 4, *n*=610, 12.1%), chronic high-impact (Group 5, *n*=84, 1.4%) (Figure 2). Chronic high-impact users had persistently high annual readmission rates, while short-term high-impact users had high annual readmission rate only during the initial period following AAA repair, and then the readmission rate declined. The covariates with positive and significant association with chronic high-impact users were ischaemic heart disease (*n*=18 (21.4%) vs. *n*=453 (10.5%), OR 2.08, CI 1.49–2.89, *P*=0.028) and socioeconomic deprivation (*n*=26 (30.9%) vs. *n*=626 (14.5%), OR 1.28, CI 1.16–1.42, *P*=0.013). The sensitivity and specificity of the model to detect chronic high-impact users were 87.2% and 99.3%, respectively. Common primary diagnoses for in-patient hospital mortality (*n*=1458) were pneumonia (*n*=276, 18.9%), cancer (*n*=212, 14.5%), heart failure (*n*=130, 8.9%), renal failure (*n*=101, 6.9%), and external injuries (*n*=71, 4.9%). Common causes of nonelective readmissions (*n*=22,705) were pneumonia (*n*=2200, 10.7%), COPD (*n*=1255, 6.1%), iatrogenic injury (*n*=931, 4.5%), urinary tract infection (*n*=897, 4.4%), and gastrointestinal haemorrhage (*n*=883, 4.3%).

**Figure 3:**
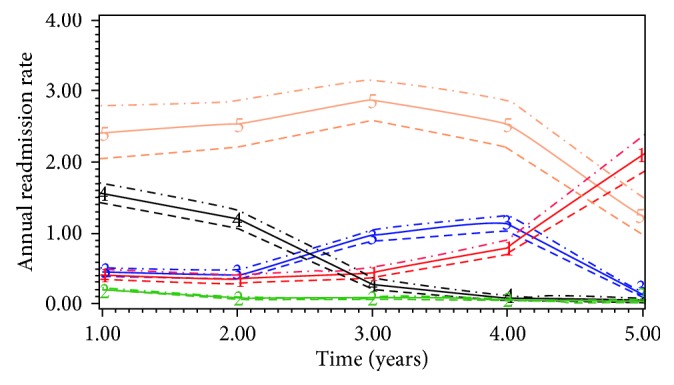
Subgroups among elective open repair patients and their trajectories of unplanned readmission rates (the horizontal axis starts with annual readmission rate after one year, and the dotted lines represent 95% confidence intervals for each subgroup).

Among elective open repair (*n*=10,801), the mean age of the patients was 72.6 (SD 7.4) years and mean LOS was 12.9 (SD 12.9) days. Only 0.5% (*n*=50) of the patients lived alone, and 0.4% (*n*=44) of them were discharged to nursing home. The 30-day and 5-year mortality rate was 6.8% (*n*=738) and 23.9% (*n*=2583), respectively. GBTM (BIC −34269, AIC −34185) classified the patient population into 5 subgroups based on their nonelective readmissions: low-impact (Group 2, *n*=8315, 71.7%), 2 intermediate groups (Group 3, *n*=1294, 13.9%; Group 1, *n*=346, 4.2%), short-term high-impact (Group 4, *n*=716, 8.9%), and chronic high-impact (Group 5, *n*=130, 1.3%) (Figure 3). The covariates with positive and significant association with chronic high-impact users were diabetes (*n*=31 (20.4%) vs. *n*=65 (8.0%), OR 2.66, 1.67–4.26, *P*=0.036) and socioeconomic deprivation (*n*=35 (26.9%) vs. *n*=1065 (12.8%), OR 1.32, CI 1.22–1.43, *P* < 0.001). Common primary diagnoses for in-hospital mortality (*n*=1940) were pneumonia (*n*=329, 16.9%), cancer (*n*=319, 16.4%), heart failure (*n*=156, 8.0%), renal failure (*n*=118, 6.1%), and metabolic/nutritional conditions (*n*=89, 4.6%). Common causes of readmissions were pneumonia (*n*=2425, 9.8%), COPD (*n*=1409, 5.7%), chest pain (*n*=1125, 4.6%), gastrointestinal haemorrhage (*n*=1099, 4.4%), and urinary tract infection (*n*=931, 3.8%).

### 3.3. Ruptured AAA Repair (rAAA)

**Figure 4:**
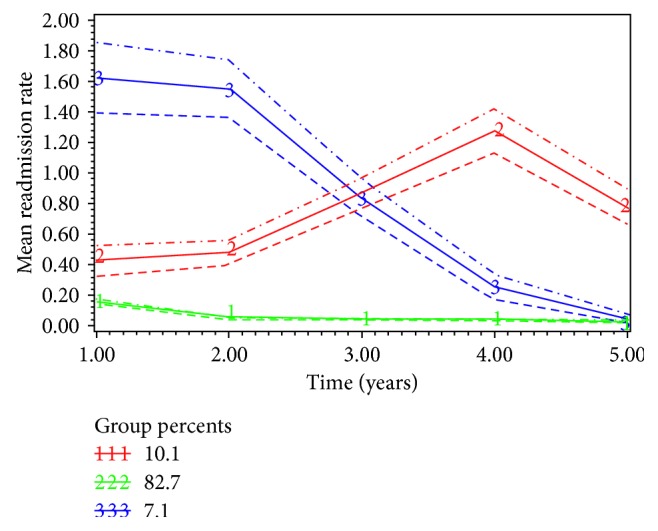
Subgroups among ruptured AAA repair patients and their trajectories of unplanned readmission rates (the horizontal axis starts with annual readmission rate at year one, and the dotted lines represent 95% confidence intervals for each subgroup).

The best-fit model (BIC −9936, AIC −9895) classified the patient population (*n*=4144) into 3 subgroups based on their nonelective annual readmission rates: Group 1 (82.7%), Group 2 (10.1%), and Group 3 (7.2%) (Figure 4). Group 1 had persistently low rate of readmission and therefore was classified as low-impact. Those with high readmission rates (high-impact users) were part of Group 2 and Group 3. Group 2 were chronic high-impact users because they had annual increase in readmission rate. Covariates associate with chronic high-impact users were renal failure, open repair, heart failure, female sex, and total number of HACs (Table 2). Group 3 were short-term high-impact users that had high readmission rate in the beginning but then had rapid decline in readmission rate. Covariates associated with short-term high-impact users were female sex, socioeconomic index, older age, and length of stay (Table 3).

The 5-year mortality rate among high-impact users, Group 2 (*n*=60, 15.0%) and Group 3 (*n*=157, 53.4%), was significantly lower than the low-impact group (*n*=1997, 57.9%, *P* < 0.001). The proportion of patients undergoing revision of procedure was higher in high-impact users, chronic (*n*=19, 4.8%) and short-term (*n*=8, 2.7%), as compared to low-impact users (*n*=23, 0.7%, *P* < 0.001). The mean number of elective vascular procedures during the follow-up period was high among high-impact users, chronic (0.12 (SD 0.59)) and short-term (0.16 (SD 0.53)), compared to the low-impact users (0.03 (SD 0.21)). Similarly, the mean number of elective admissions during the follow-up period was increased among the high-impact users, chronic (3.5 (SD 5.3)) and short-term (3.0 (SD 5.7)) as compared to low-impact users (0.9 (SD 2.2), *P* < 0.001). The sensitivity and specificity of the model to detect chronic high-impact users in the original cohort of patients was 23.5% and 94.3%, respectively. The predictors that were significantly associated with high-impact users were applied to a different cohort of patients, validation set, who were diagnosed in the year 2003-2004 for validation of the model. The AUC for c-statics was 0.71.

Most common causes of readmissions over 5-year period were not related to AAA repair. Emergency admissions due to AAA graft complications were only 2.4% (*n*=36). Other common causes of readmissions were respiratory tract infection (*n*=127, 8.4%), COPD (chronic obstructive pulmonary disease) (*n*=80, 5.3%), hypotension (*n*=77, 5.1%), gastrointestinal haemorrhage (*n*=70, 4.6%), and chest pain (*n*=70, 4.6%).

### 3.4. Sensitivity Analysis for Repair of rAAA

**Figure 5:**
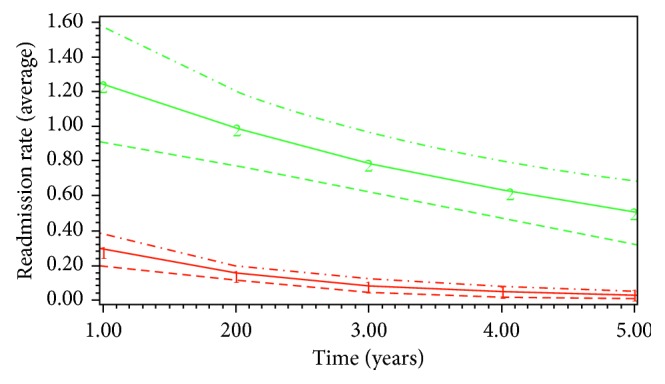
Subgroups among ruptured AAA patients with EVAR repair and their trajectories of unplanned readmission rates (the horizontal axis starts with annual readmission rate at year one, and the dotted lines represent 95% confidence intervals for each subgroup).

Among EVAR patients for rAAA (*n*=267), the mean age of the patients was 75.9 (SD 7.5) and mean LOS for the index admission was 13.2 (SD 14.2) days. Only 0.8% (*n*=2) of the patients lived alone, and 2.6% (*n*=7) of them were discharged to a nursing home. The 5-year overall mortality rate was 49.4% (*n*=132). GBTM (BIC −869, AIC −880) classified the patient population into 2 subgroups based on their emergency readmissions: low-impact (*n*=203, 78.1%) and chronic high-impact (*n*=64, 21.9%) (Figure 5). The covariates with positive and significant association with chronic high-impact users were diabetes (*n*=11 (18.3%) vs. *n*=13 (6.3%), OR 19.11, 95% CI 5.87–62.18, *P*=0.012) and prolonged LOS (mean = 18.41 (SD 19.8) vs. 11.72 (12.2), OR 2.44, 95% CI 1.82–3.25, *P*=0.002). The sensitivity and specificity of the model to detect chronic high-impact users were 100% and 81.2%, respectively. The 5 most common causes of emergency readmissions (*n*=720) were COPD (*n*=80, 13.5%), iatrogenic complications (*n*=52, 8.7%), bleeding (*n*=38, 6.4%), chest infection (*n*=37, 6.2%), and dementia (*n*=12, 3.5%). Common causes of in-hospital mortality (*n*=60) were heart failure (*n*=7, 11.7%), fractures (*n*=6, 10.0%), ruptured AAA (*n*=5, 8.3%), external injuries (*n*=5, 8.3%), cancer (*n*=4, 6.7%), pulmonary embolism (*n*=4, 6.7%), renal failure (*n*=4, 6.7%), chest infection (*n*=4, 6.7%), and stroke (*n*=4, 6.7%).

**Figure 6:**
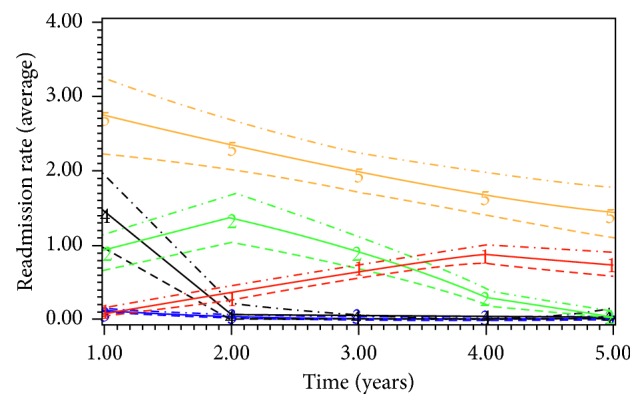
Subgroups among ruptured AAA patients with open repair and their trajectories of unplanned readmission rates (the horizontal axis starts with annual readmission rate at year one, and the dotted lines represent 95% confidence intervals for each subgroup).

Among patients with open repair for rAAA (*n*=3877), the mean age of the patients was 72.65 (SD 7.39) and mean LOS for the index admission was 12.99 (SD 12.95) days. Only 0.5% (*n*=21) of the patients lived alone, and 1.0% (*n*=39) of them were discharged to nursing home. The 5-year overall mortality rate was 53.7% (*n*=2081). GBTM (BIC −8973, AIC −8916) classified the patient population into 5 subgroups based on their emergency readmissions: low-impact (Group 3, *n*=3168, 71.7%), 2 intermediate groups (Group 2, *n*=261, 13.9%; Group 1, *n*=302, 4.2%), short-term high-impact (Group 4, *n*=97, 8.9%), and chronic high-impact (Group 5, *n*=49, 1.23%) (Figure 6). The covariate with positive and significant association with chronic high-impact users was heart failure (*n*=7 (14.9%) vs. *n*=129 (10.9%), OR 3.74, CI 2.12–6.62, *P*=0.023). The sensitivity and specificity of the model to detect chronic high-impact users were 100% and 98.89%, respectively. The five most common causes of readmissions were chest infection (*n*=705, 12.2%), COPD (*n*=411, 7.1%), gastrointestinal haemorrhage (*n*=248, 4.3%), urinary tract infection (*n*=229, 3.9%), and low blood pressure (*n*=226, 3.9%). Common primary diagnoses for in-hospital mortality (*n*=611) were chest infection (*n*=115, 19.1%), cancer (105, 17.4%), ruptured AAA (*n*=29, 4.8%), renal failure (*n*=52, 8.6%), and heart failure (*n*=53, 8.8%).

## 4. Discussion

Two subgroups were identified among patients with elective AAA repair: low- and high-impact users. Elderly female patients with poor socioeconomic status and increased comorbidities who had undergone endovascular elective repair with prolonged length of stay as well as other vascular procedures seem to be high-impact users with persistently high long-term readmission rate. There were 3 distinct groups among patients with rAAA repair. Majority of the patients were low-impact users with persistently low readmission rate. There were 2 kinds of high-impact users, one with constantly high readmission rate, chronic high-impact, and the other was, short-term high-impact, who had high readmission rate initially and then a sharp decline. Chronic high-impact users were significantly associated with renal failure, gastrointestinal complications, heart failure, peripheral vascular disease, number of hospital acquired complications, female sex, and undergoing other related procedures. High-impact users in both patient populations were also associated with higher rates of elective procedures, vascular procedures, and revision procedures. The coding system did not provide further details on the types of revision of procedure conducted on the AAA repair patients.

With the primary focus on the patients with aneurysm repair and observing them with systematic and long-term follow-up, various significant predictors associated with high-impact users were identified which were not shown to have any significant effect on the readmission rate in the earlier studies [1, 20, 22, 23]. It was important to include various types of risk factors in the model and identifying factors with strong predictive ability. Very few studies have assessed predictors and causes of increased readmission rate, especially those related to high-impact users among vascular patients [3]. In earlier studies, the most powerful predictors of readmissions in general surgical population were postoperative complications, polypharmacy, and comorbidity score. In addition, diabetes and heart failures were significantly associated with vascular surgery patients [3]. But the patients were followed up for a limited time, and no effort was made to identify and select high-impact users [24].

Certain risk factors which may be assumed to cause an increase in the readmission rate had lower odds of being associated with high-impact users like patients with a history of cardiac arrest, heart failure, and renal failure following elective AAA repair. It may suggest that patients with these risk factors had very poor prognosis and died early in the follow-up without having many readmissions. Hence, patients with increased number of comorbidities and high mortality rate were not part of high-impact group. These patients may have been frail; however, in the current coding system, frailty is not coded. On the other hand, high-impact users were relatively stable patients who survived multiple hospital admissions.

In this study, the impact of various risk factors was assessed to identify those with long-lasting effects on the readmission rates. These risk factors had been previously studied [4, 7]. Some factors were specific to the AAA repair such as other vascular procedures. These included radiological or operative interventions for vascular conditions of limbs and mesenteric vessels. The purpose to include this risk factor was to ascertain if the AAA repair was complicated by thromboembolic event in other vessels that required intervention. This would mean prolonged hospital stay for the patient and poor recovery [1, 20]. In addition, to individual cardiovascular risk factors, the cumulative effect of chronic illnesses in the patient was also assessed by using the Charlson comorbidity score. This was used to assess the burden of past medical problems on the patient. The score is based on the number of particular chronic medical conditions that the patient suffers from [7]. In this manner, the study was able to identify particular patient-based, procedure-related, and collective effect of other past medical illnesses that would make a patient prone to high readmission rate.

Our study showed that high-impact users continued to have high readmission rate during the 5-year period following AAA repair. It is important to recognise them early and assess the option of different care pathway to allow easy transition of care. The predictive model can be used to identify potential high-impact users by the surgical team. These patients may benefit from closer surveillance and prompt more aggressive pre- or postoperative cardiopulmonary work-up or rehabilitation to avoid iatrogenic complications. An early and aggressive mobility and cardiopulmonary rehabilitation program for patients in ICU and colonic surgery patients have already shown to reduce readmission rate [25, 26].

Unlike previous studies, our analysis indicated that the high-impact users following rAAA repair consisted of two subgroups. Short-term high-impact group was characterised by elderly female patients belonging to poor socioeconomic class who had prolonged length of stay. It implies that prolonged length of stay among elderly female patients makes one prone to iatrogenic complications [27, 28]. Their health status may deteriorate during their stay in the hospital which makes them prone to further earlier readmissions [29]. However, if an elderly female patient with other comorbidities undergoes open repair, it causes huge insult to body physiology leading to other complications and resulting in continuously high readmission rate, making them chronic high-impact users.

The sensitivity analysis of EVAR and open repair suggested that more than 2 subgroups exist in a patient population than previously known [7]. Traditionally, the population was divided into 2 groups, low- and high-impact users by arbitrary classification [7]. With the use of GBTM that assesses long-term trends of readmissions, patient population consisted of more than 2 subgroups with discrete trends in readmission rates. Other identified groups were intermediate and short-term high-impact users. We may assume that short-term high-impact users were those elderly patients who could not withstand multiple readmissions and die as a result of it. It will be interesting to evaluate factors associated with intermediate groups as they form a large proportion of patient population and have a tendency of having a rise in the annual readmission rate during the long-term follow-up. The common causes of long-term readmissions and mortality were similar among populations of EVAR and open repair, and they were not related to AAA repair in majority of the cases [30]. The causes were related to cardiovascular conditions, common respiratory and urine infections, falls and fractures, and cancer [30]. These conditions generally prevalent in the community and require holistic care of the patient rather than specific review the specialist vascular team [30]. Among patients with rAAA, mortality due to rAAA was also common, and this is well documented in previous clinical trials [1, 30]. Interestingly, mortality due to cancer was also common among AAA patients. Previous clinical studies have not identified any association between cancer and AAA; however, coincidental finding of cancer among AAA patients as a part of ageing population have been mentioned earlier [30, 31].

There were certain limitations associated with the study. Firstly, the identification of the patient cohort and the covariates is based on ICD and OPCS coding, which are prone to coding errors [32]. Secondly, selection of the cases in a retrospective cohort study may lead to a degree of selection bias. Thirdly, the trajectories are produced based on a single outcome, which may not provide a complete picture of use of other hospital care services. Fourthly, the administrative data also did not include information on the anatomy of abdominal aortic aneurysm, which is an important determinant of a patient's morbidity [33]. Fifthly, there may be selection bias in patients undergoing EVAR because the procedure is used for the patients with particular anatomy to fit the stent and those patients with increased comorbidities [1]. Therefore the differences between the outcomes of EVAR and open AAA repair may be accounted for by this bias.

The trajectory analysis used in the study had strong predictive power and increased specificity to identify high-impact users. The methodology to classify high-impact users was different from the ones in previously conducted studies. The model used in this study estimated a pattern of annual readmission rate for each patient and grouped patients with similar trends. The narrow confidence interval for each group showed that they were discrete. Very few earlier studies have made an effort to identify high-impact users and assess their pattern of readmission following repair for AAA [34]. In most studies, the readmission rate was measured as a secondary outcome and only for a period of 30 days to 1 year [34]. Clinical studies have recognised high-impact users among general patient population and attempted to find their risk factors. However, little is known what happens to these patients once they are labelled as high-impact users. Earlier study has shown that half of the high-impact users become low-impact after one year of follow-up. It is important to observe these patients for a long period because a significant number of emergency readmissions occur after 30 days [34].

In conclusion, group-based trajectory analysis of the patient population following AAA repair had categorised those patients with high readmission rate and visualised their pathway of long-term readmission rate. The analysis of the long-term readmission rate showed that more than 2 groups exist in addition to those previously known, low- and high-impact users. The number of groups within a patient population differs depending on their main diagnosis and treatment. The long-term readmission rate and mortality were similar in EVAR and open repair patients despite obvious differences in treatment pathways. Majority of the causes of readmissions and mortality were not related to AAA repair. The factors associated with high-impact users may highlight in which areas improvements in perioperative care must be set. The results of the study imply that there should be a change in the strategy of long-term management of AAA patients who survive the perioperative period. Predictors associated with high-impact users help clinicians to identify those who have the tendency to become high-impact users. Clinicians can concentrate on the management of specific subgroups with higher readmission rates and limit elaborate spending of resources on a large group of patients that are well and will continue to be so.

## Figures and Tables

**Table 1:** Covariates associated with high-impact users compared to low-impact users among patients who had elective AAA repair.

Risk factors	Low-impact users	High-impact users	Odds ratio (95% CI)	*P* value
*Patient demographics*				
Living alone	49 (0.3)	24 (0.8)	1.65 (1.21–2.25)	0.11
Female sex	1829 (13.1)	509 (16.9)	1.23 (1.15–1.32)	**0.001**
Socioeconomic index	2.8 (1.3)	3.1 (1.3)	1.13 (1.11–1.15)	**<0.001**
Age	73.1 (SD 7.3)	75.6 (SD 7.1)	1.04 (1.04–1.04)	**<0.001**
Non-Caucasian ethnicity	2081 (14.9)	297 (9.9)	0.61 (0.57–0.66)	**<0.001**

*Past medical history*				
Diabetes	1477 (10.6)	409 (13.6)	1.22 (1.07–1.39)	0.11
Anaemia	350 (2.5)	105 (3.5)	1.21 (1.06–1.37)	0.17
Ischaemic heart disease	1478 (10.6)	414 (13.8)	1.15 (1.07–1.23)	0.06
Mental health disorders	169 (1.2)	55 (1.8)	1.12 (0.91–1.36)	0.57
Hypotension	232 (1.7)	59 (1.9)	1.05 (0.88–1.26)	0.76
Comorbidity score	3.9 (SD 5.7)	5.6 (SD 6.6)	1.04 (1.04–1.05)	**<0.001**
Stroke	202 (1.4)	85 (2.8)	0.98 (0.83–1.16)	0.92
Hypertension	7556 (64.1)	1645 (54.7)	0.97 (0.92–1.02)	0.47
Chronic peripheral vascular disease	671 (4.8)	161 (5.4)	0.80 (0.72–0.90)	**0.05**
Heart failure	435 (3.1)	125 (4.2)	0.59 (0.51–0.68)	**<0.001**

*Management factors*				
Urinary tract infection	272 (1.9)	79 (2.6)	1.31 (1.08–1.58)	0.17
Wound infection	316 (2.3)	80 (2.7)	1.22 (1.04–1.42)	0.19
Other vascular procedures	3842 (27.5)	1167 (38.8)	1.21 (1.14–1.28)	**0.003**
Revision of graft	32 (0.2)	9 (0.3)	1.21 (0.81–1.80)	0.67
Other graft complications	287 (2.1)	89 (2.9)	1.12 (0.96–1.30)	0.46
Respiratory tract infection	1083 (7.7)	228 (7.6)	1.01 (0.87–1.17)	0.94
Prolonged length of stay	10.6 (SD 11.6)	11.5 (SD 12.7)	1.00 (1.00–1.01)	**<0.001**
Wound dehiscence	589 (4.2)	133 (4.4)	0.97 (0.86–1.09)	0.75
Total number of HACs	0.24 (0.51)	0.27 (0.53)	0.92 (0.83–1.03)	0.42
Acute peripheral vascular disease	337 (2.4)	78 (2.6)	0.92 (0.79–1.07)	0.59
Other procedures	733 (5.2)	135 (4.5)	0.87 (0.77–0.98)	0.23
Pulmonary embolism/deep venous thrombosis	56 (0.4)	7 (0.2)	0.87 (0.52–1.45)	0.78
Gastrointestinal complications	571 (4.1)	106 (3.5)	0.83 (0.73–0.94)	0.13
Other procedural complications	150 (1.1)	27 (0.9)	0.78 (0.60–1.01)	0.31
Discharge to nursing home	53 (0.4)	12 (0.4)	0.76 (0.52–1.12)	0.47
Graft infection	56 (0.4)	11 (0.4)	0.73 (0.49–1.11)	0.44
Renal failure	508 (3.6)	115 (3.8)	0.72 (0.63–0.82)	**0.01**
Open AAA repair	9292 (66.5)	1509 (50.2)	0.61 (0.58–0.65)	**<0.001**
Cardiac arrest	148 (1.1)	9 (0.3)	0.26 (0.17–0.39)	**<0.001**

**Table 2:** Covariates associated with Group 1 (chronic high-impact) as compared to low-impact users among patients who had rAAA repair.

Risk factors	Low-impact (*n* (%)) or mean (SD)	Chronic high-impact (*n* (%)) or mean (SD)	OR	CI (95%)	*P* value
*Patient demographics*					
Age	73.5 (SD 7.6)	75.5 (SD 7.5)	1.01	0.99–1.03	0.51
Female sex	283 (12.5)	91 (19.4)	3.03	2.05–4.48	**0.005**
Non-Caucasian ethnicity	332 (14.6)	326 (23.2)	1.58	1.09–2.29	0.21
Socioeconomic index	2.9 (SD 1.3)	2.9 (SD 1.3)	0.98	0.88–1.10	0.88

*Past medical history*					
Comorbidity score	4.1 (SD 6.1)	5.8 (SD 8.4)	0.98	0.96–1.00	0.58
Heart failure	92 (4.1)	120 (8.5)	7.77	3.39–17.81	**0.01**
Peripheral vascular disease	164 (7.2)	143 (10.2)	7.24	4.06–12.94	**<0.001**
Mental health disorders	37 (1.6)	22 (1.6)	1.19	0.32–4.44	0.89
Diabetes	177 (7.8)	147 (10.5)	1.15	0.63–2.09	0.82
Ischaemic heart disease	293 (12.9)	169 (12.0)	1.06	0.68–1.65	0.88
Hypertension	1127 (49.7)	567 (40.3)	0.73	0.54–0.97	0.26

*Procedural characteristics*					
Open repair	2095 (92.4)	1358 (96.6)	7.85	4.41–13.96	**<0.001**
Other vascular procedures	394 (17.4)	187 (13.3)	0.47	0.31–0.71	0.07
Other procedures	340 (13.9)	261 (18.6)	2.64	1.62–4.31	**0.05**
Admission to ITU	82 (8.9)	126 (3.0)	1.93	1.06–3.53	0.27
Length of stay	23.7 (SD 21.7)	3.4 (SD 4.2)	0.59	0.55–0.62	**<0.001**

*Procedure-related complications*			
Renal failure	298 (13.1)	261 (18.6)	11.59	6.89–19.49	**<0.001**
Gastrointestinal complications	233 (10.3)	103 (7.3)	3.32	1.79–6.17	**0.05**
Graft complications	73 (3.2)	25 (1.8)	2.01	0.48–8.41	0.62
Wound complications	371 (16.4)	234 (16.6)	1.86	1.19–2.92	0.17
Hypotension	83 (3.7)	85 (6.0)	1.27	0.61–2.66	0.74
Anaemia	100 (4.4)	35 (2.5)	0.88	0.34–2.27	0.88

*Hospital acquired complications*				
Total number of HACs	0.51 (SD 0.75)	0.42 (SD 0.64)	2.56	1.77–3.71	**0.01**
Respiratory tract infection	544 (23.9)	217 (15.4)	1.86	1.02–3.39	0.29
Urinary tract infection	89 (3.9)	10 (0.7)	0.84	0.24–3.00	0.89

**Table 3:** Covariates associated with Group 3 (short-term high-impact) as compared to low-impact users among patients who had rAAA repair.

Risk factors	Low-impact (*n* (%)) or mean (SD)	Short-term (*n* (%)) or mean (SD)	OR	CI (95%)	*P* value
*Patient characteristics*					
Age	73.5 (SD 7.6)	75.2 (SD 7.5)	1.02	1.02–1.03	0.005
Female sex	283 (12.5)	91 (19.4)	1.48	1.26–1.73	**0.02**
Non-Caucasian ethnicity	332 (14.6)	78 (16.6)	1.13	0.96–1.33	0.46
Socioeconomic index	2.9 (SD 1.3)	3.2 (SD 1.3)	1.16	1.11–1.22	**0.001**

*Past medical history*					
Comorbidity score	4.1 (SD 6.1)	4.8 (SD 6.9)	1.02	1.00–1.03	0.06
Peripheral vascular disease	164 (7.2)	43 (9.1)	1.16	0.92–1.46	0.53
Diabetes	177 (7.8)	43 (9.1)	1.13	0.87–1.46	0.65
Hypertension	1127 (49.7)	232 (49.4)	0.94	0.83–1.07	0.63
Heart failure	92 (4.1)	26 (5.5)	0.76	0.53–1.07	0.41
Ischaemic heart disease	293 (12.9)	48 (10.2)	0.73	0.60–0.89	0.10
Mental health disorders	37 (1.6)	7 (1.5)	0.67	0.41–1.10	0.42
Stroke	41 (1.8)	6 (1.3)	0.51	0.28–0.94	0.26

*Procedural characteristics*					
Other vascular procedures	394 (17.4)	97 (20.6)	1.07	0.90–1.29	0.69
Length of stay	23.7 (SD 21.7)	26.5 (SD 24.5)	1.01	1.00–1.01	**0.02**
Other procedures	340 (13.9)	58 (12.3)	0.89	0.73–1.08	0.54
Open repair	2095 (92.4)	424 (90.2)	0.78	0.61–0.99	0.29
Admission to ITU	82 (8.9)	11 (2.3)	0.71	0.49–1.04	0.37

*Procedure-related complications*					
Anaemia	100 (4.4)	22 (4.7)	1.08	0.82–1.43	0.77
Graft complications	73 (3.2)	15 (3.2)	0.98	0.70–1.38	0.95
Gastrointestinal complications	233 (10.3)	42 (8.9)	0.83	0.67–1.02	0.38
Wound complications	371 (16.4)	57 (12.1)	0.71	0.59–0.86	0.07
Renal failure	298 (13.1)	54 (11.5)	0.69	0.56–0.84	0.06

*Hospital acquired complications*					
Urinary tract infection	89 (3.9)	23 (4.9)	1.38	0.98–1.93	0.34
Respiratory tract infection	544 (23.9)	119 (25.3)	1.12	0.89–1.41	0.62
Total number of HACs	0.51 (SD 0.75)	0.53 (SD 7.9)	0.95	0.82–1.11	0.74
Hypotension	83 (3.7)	10 (2.1)	0.56	0.37–0.84	0.16

## Data Availability

The data used to support the findings of this study are available from the corresponding author upon request.
